# A Case of Diabetes With a Rare Variant of Familial Combined Hyperlipidemia With an Atypical Phenotype

**DOI:** 10.7759/cureus.85263

**Published:** 2025-06-02

**Authors:** Karishma Bhade, Nimisha Dange, Vaman Khadilkar, Anuradha Khadilkar

**Affiliations:** 1 Growth and Pediatric Endocrinology, Hirabai Cowasji Jehangir Medical Research Institute, Pune, IND; 2 Health Sciences, Savitribai Phule Pune University, Pune, IND

**Keywords:** diabetes, dyslipidemia, familial combined hyperlipidemia (fch), lipodystrophy, lpl

## Abstract

A female patient from India was diagnosed with diabetes at 16 years of age and presented to a tertiary care diabetes clinic nine months later with hyperglycemia and dyslipidemia (hypercholesterolemia and hypertriglyceridemia), while on insulin and statin therapy. The striking feature was the presence of lipodystrophy on both her upper and lower limbs. Both her elder brother and mother showed the presence of dyslipidemia and a normal phenotype on cascade screening. All three of them had a normal BMI. Managing the case was challenging due to the suboptimal response of dyslipidemia to the various combinations of medication. Genetic testing revealed a rare mutation in the LPL gene, causing familial combined hyperlipidemia (FCH) with an unusual association of lipodystrophy. A similar heterozygous mutation was found in the mother. We report the first case of FCH with lipodystrophy from India and share the challenges encountered during the three years of follow-up.

## Introduction

Familial combined hyperlipidemia (FCH; Online Mendelian Inheritance in Man or OMIM 144250) is a common cause of primary dyslipidemia with an estimated prevalence of 0.5%-4% [1]. It is characterized by high serum cholesterol and/or triglyceride, apolipoprotein B, atherogenic small dense low-density lipoprotein (LDL) particles and decreased levels of high-density lipoprotein cholesterol (HDL-C) in at least two members of the family [2]. The condition was initially described as an autosomal dominant disorder, and was later found to have a complex multigenic inheritance [3,4]. FCH has a polygenic inheritance, and several genes and genetic variants have been associated with its pathogenesis. Some of these include upstream transcription factor-1 (USF1), lipoprotein lipase (LPL), low-density lipoprotein receptor (LDLR), proprotein convertase subtilisin kexin type-9 (PCSK9), etc. [4].

FCH shows variable expression and penetrance, with onset and symptoms differing among individuals within the same family. Patients may experience chest discomfort, breathing difficulty, or leg pain. Physical examinations are typically normal; rarely, xanthomas may be detected [2].

We present a three-year follow-up of a non-obese Indian adolescent female patient with type 2 diabetes, insulin resistance (IR), lipodystrophy and genetically confirmed FCH. This rare genotype-phenotype correlation appears to be unreported in the literature.

## Case presentation

**Figure 1:**
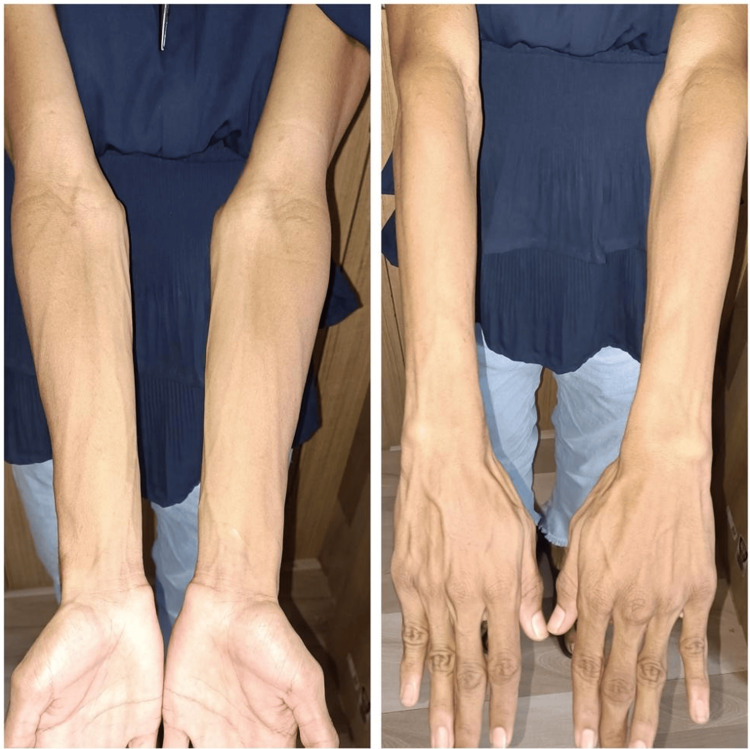
Upper limb lipodystrophy and phlebomegaly

A female patient, who was 16 years and nine months old and the second child born of a non-consanguineous union, was referred to our clinic for diabetes, diagnosed nine months ago. She was born by normal vaginal delivery at term and weighed 2.5 kg (-0.82 standard deviation or SD). At 16 years of age, she developed polyuria, polydipsia, fatigue, and lethargy. She was started on a basal-bolus insulin regimen and statin therapy for severe dyslipidemia diagnosed during screening. On examination, she was normotensive with a height of 158.2 cm (0.1 SD), weight of 39.9 kg (-1.4 SD), BMI of 15.9 kg/m² (-1.6 SD), and waist circumference of 67 cm (-1.9 SD). She was tall for mid-parental height (MPH) of 147.5 cm (-1.8 SD) and had adult sexual maturity (Tanner stage 5) with irregular menses for the last three months. She had grade 2 acanthosis over the neck/axilla and pronounced loss of subcutaneous fat over the abdomen, gluteal region, and limbs (Figure 1).

Her C-peptide was 7.85 ng/ml with very high HbA1c of 16.9% at presentation. Her triglyceride was >10000 mg/dL, very low-density lipoprotein (VLDL) was >2000 mg/dl with other lipid parameters also being deranged (total cholesterol 1160 mg/dL, LDL-C 353 mg/dL). Her baseline and follow up lipid profile, HbA1c, and ongoing treatment are illustrated in Table 1.

**Table 1:** Investigations and treatment over the three years of follow-up *The double dashes are used to distinguish the morning and evening doses. HbA1c: Glycated hemoglobin; HDL-C: High-density lipoprotein cholesterol; LDL-C: Low-density lipoprotein cholesterol; VLDL-C: Very low-density lipoprotein cholesterol; ACR: Albumin-creatinine Ratio; TDD: Total daily dose; BD: Twice daily; OD: Once daily

Laboratory parameters	Conventional unit	Reference values in conventional units	Measured values at different time points (age of the patient)											
-	-	-	16 years	16.9 years	17 years	17.2 years	17.5 years	17.9 years	18.3 years	18.6 years	18.9 years	19.1 years	19.5 years	19.9 years
HbA1c	%	<5.7	16.1	10.1	-	5.6	6.1	8	6	5.9	6.7	7.3	12.6	7.8
Total cholesterol	mg/dl	<160	1160	280	103.5	-	-	231	210	236	223	149	253	197
Triglycerides	mg/dl	<110	>10000	1705	223	-	-	2656	1989	2197	2075	2738	5023	4270
HDL-C	mg/dl	>60	10	28	18.4	-	-	15	15	18	14	16	13	11
LDL-C	mg/dl	<100	353	-	40.4	-	-	48	46	49	44	33	33	27
VLDL-C	mg/dl	-	>2000	341	44.6	-	-	533	397	439	415	547	1005	854
Apolipoprotein A1	mg/dl	80-175	-	82	-	-	-	-	-	-	-	-	65	-
Apolipoprotein B	mg/dl	<130	-	98	-	-	-	-	-	-	-	-	85	-
Amylase	U/lt	40-140	-	-	-	-	-	-	-	-	-	30	-	-
Lipase	U/lt	10-150	-	-	-	-	-	-	-	-	-	48	-	-
C-peptide	ng/ml	0.9-1.8	-	-	-	-	-	-	7.85	-	-	-	2.84	-
Urine ACR	mg/g	<30	-	25.7	-	-	-	17.85	175	215	179	-	-	83.6
Treatment*	-	-	Basal bolus Insulin (Glargine 16U and Regular) regimen (TDD-1U/kg/day)	Basal bolus Insulin (Glargine 16U and Regular) regimen (TDD-1U/kg/day)	Basal bolus Insulin (Glargine 16U and Regular) regimen (TDD-1U/kg/day)	Basal bolus Insulin (Glargine 16U and Regular) regimen (TDD-1U/kg/day)	Basal bolus Insulin (Glargine 16U and Regular) regimen (TDD-1U/kg/day)	Glargine 16U	Glargine stopped	-	-	Degludec 16 U	Degludec 28U	Glargine 25U
	-	-	Rosuvastatin 10 mg OD	Rosuvastatin 10 mg OD	Rosuvastatin stopped	-	-	Metformin 500 mg BD, Tab Gemfibrozil 300 mg BD	Metformin 1g BD, Tab Gemfibrozil 600-300 mg BD	Metformin 1g--500mg mg BD, Tab Gemfibrozil -600--300 mg BD, Tab Ezetimibe 10 mg, Tab Envas 2.5 mg BD	Metformin 1000--500 mg BD, Gemfibrozil 600--300 mg BD, Tab Envas 2.5 mg BD	Metformin 1000--500 mg BD, Gemfibrozil 300--300 mg BD, Tab Envas 2.5 mg BD, Tab Atorvastatin 20 mg OD, Tab Finofibrate 160 mg	Metformin 500--500 mg BD, Gemfibrozil 300--300 mg BD, Tab Envas 2.5 mg BD, Tab Atorvastatin 10 mg OD, Tab Daonil 5 mg BD, Tab Saroglitazar 4 mg OD	Gemfibrozil 300--300 mg BD, Tab Envas 2.5 mg BD, Tab Atorvastatin 10 mg OD, Tab Daonil 5 mg TDS, Tab Saroglitazar 8 mg OD, Tab Pioglitazone 15 mg OD, Tab Metformin 500 mg BD

Her liver function test and pancreatic enzyme levels were normal. Her sugars and lipids responded well to basal-bolus insulin regimen and statins. However, after an year of treatment, she was lost to follow up. She stopped the statin on her own and the insulin was continued. She returned after an year at 17.9 years of age with multiple hypoglycemic episodes and a severely deranged lipid profile. Her bolus Insulin was stopped and basal insulin was continued. Metformin therapy was started due to signs of Insulin resistance (acanthosis and irregular menses) and gemfibrosil was started for managing the very high triglycerides and cholesterol. She developed diabetic nephropathy with normotensive BP readings. Her urine microalbumin-to-creatinine ratio (UACR) was 175 mg/g and 215mg/g at two different occasions three months apart. So, enalapril was started, to which she responded well (repeat UACR after one year of treatment was 83.7 mg/g). As her glycemic control was satisfactory (HbA1c reduced from 10.1% to 6%), basal insulin was stopped but dyslipidemia (high triglycerides, total and VLDL cholesterol, low HDL-C) showed unsatisfactory response to fibrates, statins as well as to ezetimibe. Her sugar levels on self-monitoring of blood glucose (SMBG) started rising and thus basal insulin had to be restarted. However, her HbA1c worsened from 7.3% to 12.6%. Despite insulin therapy, her HbA1c increased to 12.3%. This was also partially due to non-compliance to insulin. To facilitate endogenous insulin production and considering the beneficial effect of combination therapy with metformin and glibenclamide, the decision to add glibenclamide to her treatment plan was made. Pioglitazone and saroglitazar were added subsequently for due to their dual action on hyperglycemia as well as dyslipidemia. Though the hyperglycemia control was fairly adequate initially, all the lipid parameters remained deranged (Table 1). Ultrasound doppler for carotid intima media thickness was normal (right-0.54 mm, left-0.46 mm) and that of the pancreas showed no abnormality. On cascade screening, both her mother and elder brother were also found to have dyslipidemia (Table 2).

**Table 2:** Cascade screening

Lab parameters	Reference values in conventional units	Results	
		Mother (45 years)	Elder brother (25 years)
Total Cholesterol (mg/dL)	<200	206	220
Sr. Triglyceride (mg/dL)	<150	199.8	415
HDL (mg/dL)	>40	37.2	28.3
LDL (mg/dL)	<100	129.4	109
VLDL (mg/dL)	-	39.9	83
HbA1c (%)	<5.6	5.6	5.3

**Figure 2:**
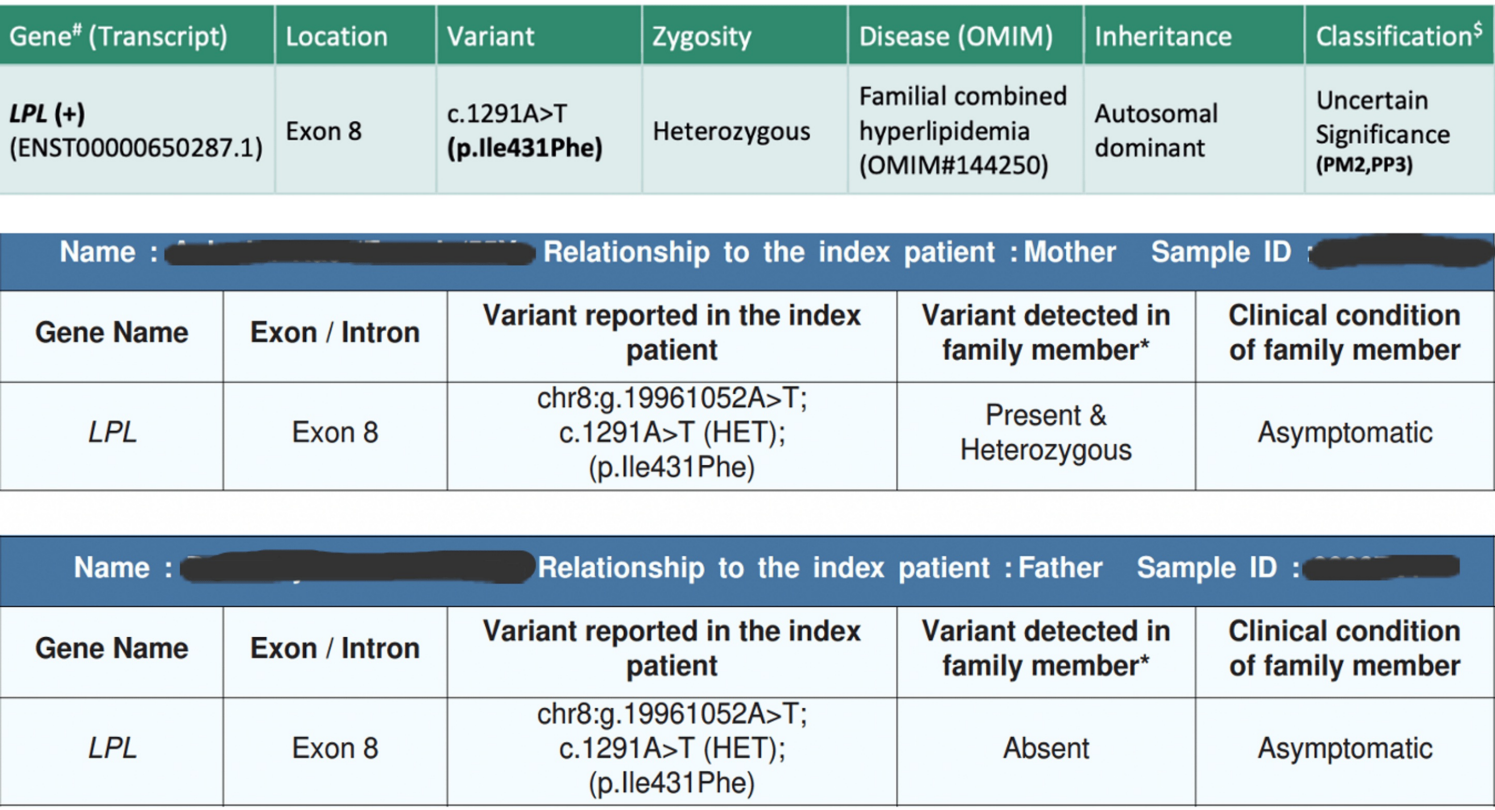
Whole-exome sequencing report of the index patient and the clinical exome reports of her parents

**Figure 3:**
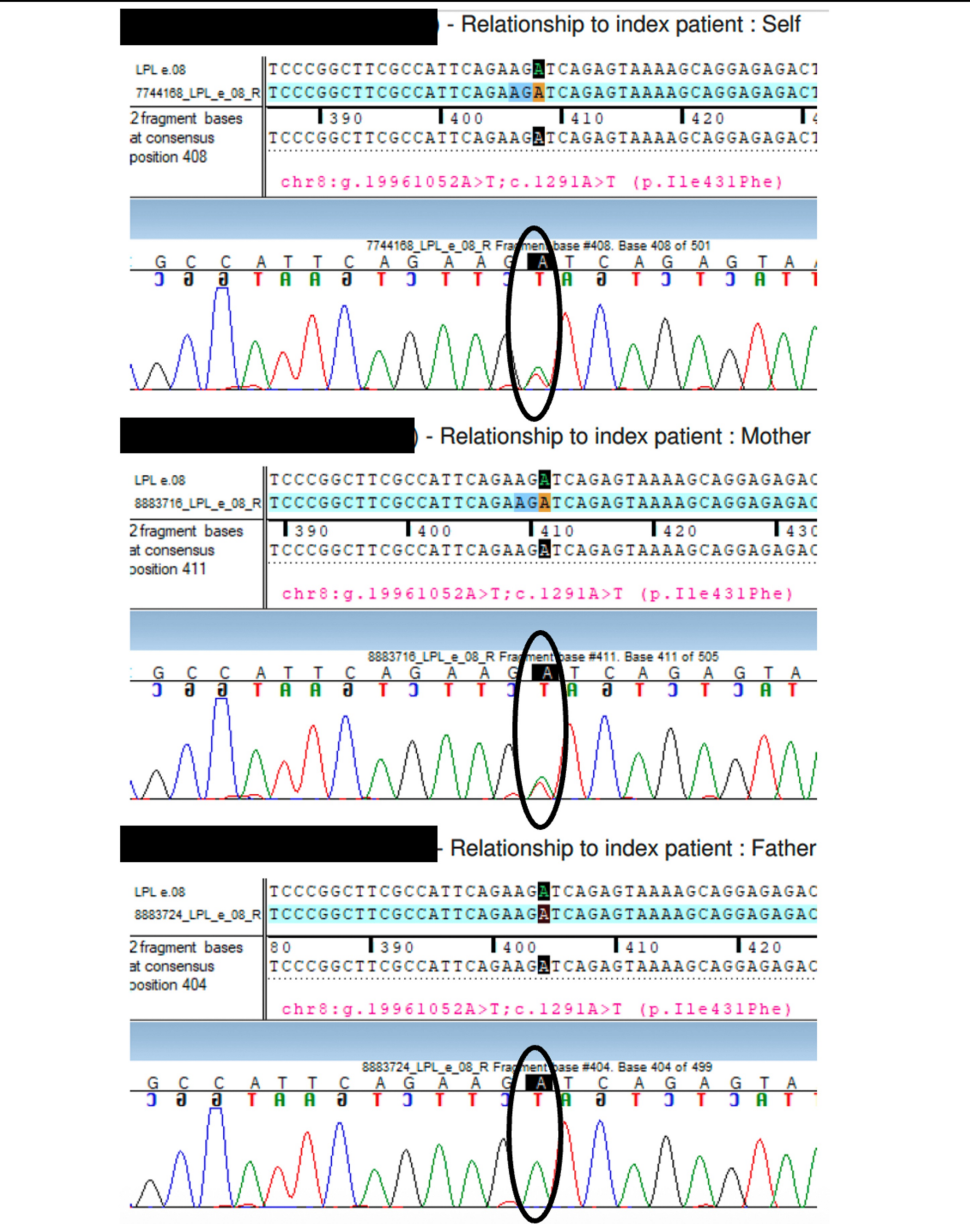
Sanger sequence chromatogram and alignment to the reference sequence It shows the variation in exon 8 of the LPL gene (chr8:g.19961052A>T; c.1291A>T) detected in the heterozygous state in the index case (self) and the mother, but not in the father.

In view of her lipodystrophic changes, a dual-energy X-ray Absorptiometry (DXA) scan was planned to look for body composition parameters. It was done twice at presentation and at the two-year follow-up, which demonstrated a low total fat mass of 11.6% and 12.4% with a relatively high total lean mass of 84.2% and 83.8%, respectively. Whole-exome sequencing (WES; MedGenome Clinical Genomics Team, MedGenome Labs Ltd., Bangalore, India) ) was ordered in view of refractory dyslipidemia, unusual phenotype and dyslipidemia in two other non-obese and non-diabetic family members. It revealed a heterozygous missense variant in exon 8 of LPL gene that resulted in amino acid substitution of phenylalanine with isoleucine at codon 431 causing FCH. We tested both parents for this gene and the mother (who is underweight with a BMI of 15.9 kg/m^2^ without lipodystrophy) was found to carry the same heterozygous mutation in the LPL gene (Figures 2, 3).

## Discussion

An adolescent diagnosed with type 2 diabetes mellitus, dyslipidemia and lipodystrophy at 16 years, was followed up for three years. Her blood sugar showed an excellent response to a basal bolus insulin regimen initially. She was then shifted to an oral hypoglycemic agent, but continued to require basal insulin despite multiple oral hypoglycemic agents. She developed nephropathy and hypertension, which responded well to the treatment (Table 1). Her cholesterol responded modestly to statins, while triglycerides showed a limited response to fibrates and none to ezetimibe. Despite prolonged dyslipidemia, she did not develop pancreatitis. There was no evidence of xanthoma/xanthelasma, although she did have lipodystrophy. Familial dyslipidemia and partial lipodystrophy syndrome were our differential diagnoses. 

WES revealed a novel heterozygous missense variant at exon 8 of the LPL gene (c.1291A>T(p.Ile431Phe)), causing substitution of phenylalanine for isoleucine at codon 431. A total of 428 target regions were analyzed. The depth of the coverage was >80-100, and the average on-target depth was 153.96. The Combined Annotation Dependent Depletion (CADD version 1.6, MedGenome Clinical Genomics Team, MedGenome Labs Ltd., Bangalore, India) score was 23.68 (likely pathogenic), the Rare Exome Variant Ensemble Learner (REVEL, MedGenome Clinical Genomics Team, MedGenome Labs Ltd., Bangalore, India) score was 0.58 (possibly pathogenic), and the Sorting Intolerant from Tolerant (SIFT version 5.2.2, MedGenome Clinical Genomics Team, MedGenome Labs Ltd., Bangalore, India) and Polymorphism Phenotyping 2 (Polyphen version 2.2.2, MedGenome Clinical Genomics Team, MedGenome Labs Ltd., Bangalore, India) have reported this variant as damaging.

Various databases (1000 genomes, gnomAD (version 4.1) and TOPmed (MedGenome Clinical Genomics Team, MedGenome Labs Ltd., Bangalore, India) were checked, and in-silico tools (Variant Effect Predictor (version 104); SIFT; PolyPhen version 2.2.2 (likelihood ratio test (LRT) version; November 2009); CADD (version 1.6); Splice AI (Illumina, CA, US); dbNSFP version 4.2 (University of South Florida, Florida, US) and MutationTaster 2 [5]) were used. This variant is reported with allele count 4 in the South Asian population with a minor allele frequency (MAF) of 0.00004392, which categorizes it as a rare mutation. This heterozygous mutation results in FCH, and although the report classifies it as of uncertain significance, a similar mutation was found in the mother as well.

Our patient's dyslipidemia was refractory to multiple lipid-lowering drugs. Various animal studies have shown that LPL deficiency is refractory to lipid-lowering drugs as well as enzyme replacement therapy [6]. A case report from South India has reported a homozygous missense variant on exon 5 of the LPL gene (chr8:g.19954168G > C) causing Type 1 familial triglyceridemia in a seven-year-old female patient with repeated pancreatitis, who responded quite well to gemfibrosil [7]. Another case report involved two neonates, who were found to have compound heterozygous mutation (c.347G > C and c.472 T > G) and a homozygous mutation (c.836 T > G) in the LPL gene and had shown suboptimal cholesterol and triglyceride levels after long-term follow-up [8].

Though FCH is the most common form of the familial dyslipidemias, its genetic etiology is not very well understood [1,9]. Some metabolic defects like malfunctioning of adipose tissue, impaired metabolism of lipoprotein particles, reduced clearance of apolipoprotein B100 (apoB), hepatic fat accumulation, overproduction of VLDL in the liver and insulin resistance (IR) have been found to accompany FCH [10]. The adipose tissue dysfunction in FCH is well known. Arner et al. demonstrated that the turnover of triglycerides is reduced in the adipose tissue of patients with FCH [11]. The LPL enzyme is a gatekeeper enzyme for the entry and accumulation of fatty acids in tissues like adipose tissue, skeletal muscle, and cardiac muscle [12]. According to this hypothesis, reduced LPL activity in adipose tissue may limit free fatty acid (FFA) uptake and lipid accumulation. Alternatively, increased LPL expression in muscle could redirect nutrient fats from adipose tissue to muscle ("substrate steal"), thereby restricting FFA uptake in the adipocytes [13]. However, in animal studies, LPL knock out mice were found to have normal adipose tissue along with hypercholesterolemia, hypertriglyceridemia, and low HDL, suggesting the existence of an LPL-independent pathway of fatty acid generation in adipocytes. Unlike humans, this resulted in neonatal death in these mice [14]. Mice with homozygous LPL deletion were found to have low adipose tissue stores and the resulting severe hypertriglyceridemia was lethal. However, those with heterozygous deletion survive to adulthood, have mild hypertriglyceridemia and impaired VLDL clearance with normal adipose tissue [15]. There are no published case reports or studies in humans which have demonstrated lipodystrophy as one of the features resulting from this particular mutation in the LPL gene. The presence of lipodystrophy with the relatively high total lean mass in our patient, with the classical lipid phenotype of FCH and the heterozygous mutation in the LPL gene raises the suspicion of selective LPL enzyme loss in the adipose tissue.

## Conclusions

This case report presents a rare mutation in the LPL gene (exon 8, p.Ile431Phe) as the underlying cause of FCH, accompanied by an uncommon clinical association with lipodystrophy. The presence of this specific mutation is significant as it alters the function of LPL, a key enzyme in triglyceride metabolism. The coexistence of lipodystrophy, characterized by abnormal fat distribution, adds clinical complexity, suggesting a broader metabolic disruption beyond lipid abnormalities.

Management of this case proved particularly challenging due to a poor therapeutic response to conventional lipid-lowering strategies. The unpredictable progression of the disease further complicated treatment planning and patient monitoring. Long-term outcomes will depend heavily on adherence to treatment, lifestyle modifications, and close clinical follow-up. This case underscores the need for personalized therapeutic approaches and highlights the potential of genetic variants in influencing both disease expression and treatment efficacy in lipid disorders.
